# *Paepalanthus serpens*, a new microendemic species of Eriocaulaceae from the Espinhaço Range, Minas Gerais, Brazil

**DOI:** 10.3897/phytokeys.48.6713

**Published:** 2015-04-15

**Authors:** Livia Echternacht, Marcelo Trovó

**Affiliations:** 1 Instituto de Biologia, Universidade Federal de Uberlândia, Campus Umuarama, Rua Ceará s/n Bloco 2D, CEP 38400-902, Uberlândia, MG, Brazil; 2 Departamento de Botânica, Instituto de Biologia, Universidade Federal do Rio de Janeiro, Av. Carlos Chagas Filho 373, CEP 21941-590, Rio de Janeiro, RJ, Brazil

**Keywords:** *Campo Rupestre*, Microendemism, Paepalanthoideae, Poales, Threatened species

## Abstract

We describe and illustrate *Paepalanthus serpens*, a microendemic species of Eriocaulaceae from the Espinhaço Range. The species is known from a single population growing in rocky areas of the Serra do Cipó, Minas Gerais. It is placed in Paepalanthus ser. Paepalanthus, and is easily distinguished from its congeneric species by its elongated, lignescent stem, thickened by the marcescent sheaths of the linear leaves, which are arranged in a rosette at the stem apex, scapes equalling the leaf height, and capitulae with straw-coloured involucral bracts. Comparisons with the morphologically similar species are provided, as well as comments on distribution, ecology, phenology and conservation status.

## Abstract (pt)

Descrevemos e ilustramos *Paepalanthus serpens*, espécie microendêmica de Eriocaulaceae da Cadeia do Espinhaço. Esta espécie é conhecida por apenas uma população, que cresce nos campos rupestres da Serra do Cipó, Minas Gerais. A espécie é classificada em Paepalanthus ser. Paepalanthus e é facilmente distinguida das demais espécies do gênero pelo caule alongado e lignificado, espessado pelas bainhas mascescentes das folhas lineares, as quais estão dispostas em roseta no ápice do caule, escapos na altura das folhas e capítulos com brácteas estramíneas. Comparações com as espécies morfologicamente mais próximas são providas, assim como comentários sobre distribuição, ecologia, fenologia e estado de conservação.

## Introduction

*Paepalanthus* Mart. is the largest genus of Brazilian Angiosperms, comprising around 360 species in the country (Forzza et al. 2010). The genus is highly diversified in the Espinhaço Range, in both of its subunits, in Bahia and Minas Gerais states. Elevated areas in Goiás state are a secondary center of diversity in Brazil (Giulietti and Hensold 1991, Giulietti et al. 2012). As a large genus, have some authors subdivided *Paepalanthus* into many series, based mostly on rough external morphology (Koernicke 1863, Ruhland 1903). Paepalanthus ser. Paepalanthus (=Paepalanthus ser. Variabiles Ruhland) is the largest category, encompassing around 200 species (Ruhland 1903, Silveira 1908, 1928). Species placed in this series present trimerous flowers and a particular architecture, usually consisting of a short, horizontal, and subterraneous stem, rarely lignose and elongated, with congest leaves at the apex, which are mostly arranged in rosettes (Ruhland 1903). Paepalanthus ser. Paepalanthus appears polyphyletic (Andrade et al. 2010, Giulietti et al. 2012, Trovó et al. 2013), but further taxonomic and phylogenetic researches are necessary to enhance a stable classification. Therefore, assignments of species to it are possibly provisional. Recent fieldwork and herbarium studies revealed that in spite of the large number of species in this series, there are still many others to be described (e.g. Trovó et al. 2012, Trovó et al. 2013). Based on herbarium specimens and recent field efforts, we describe a remarkable new species of *Paepalanthus*, and place it within Paepalanthus ser. Paepalanthus.

## Taxonomy

### Paepalanthus serpens

Taxon classificationPlantaePoalesEriocaulaceae

Echtern. & Trovó sp. nov.

urn:lsid:ipni.org:names:77146543-1

Figures 1 , 2

#### Type.

Brazil. Minas Gerais, Santana do Pirapama, Reserva Particular do Patrimônio Natural (RPPN) Toucan Cipó, Trilha da Captação, 19°00'13.2"S, 43°45'23.3"W, SAD69, 927 m, 27 Jul 2013, *L. Echternacht, T. V. Bastos, M. Stallegger, C. A. Ferreira Júnior 2316* (holotype HUFU; isotypes BHCB, NY, P, R, SPF)

#### Diagnosis.

*Paepalanthus serpens* differs from the other species of the genus by its elongated, lignescent stem, thickened by the marcescent sheaths of the linear leaves, which are disposed in rosette at the stem apex, scapes equalling the leaf length and capitula with straw-coloured involucral bracts.

#### Description.

Perennial herbs. Stem elongate, lignescent, with a thick cover of marcescent leaf sheaths, ca. 1.0−30.0 cm long × 1.0−3.5 cm wide (without the leaf sheath coat), unbranched, pilose, with simple, filamentous trichomes ca. 1.0 cm long. Leaves arranged in rosette at the stem apex, flat to semi-terete, linear, chartaceous, 5.0−10.0 cm long × 0.3−1.5 mm wide, green, pubescent to glabrescent on both surfaces, trichomes ca. 0.1−0.2 mm long, simple, filamentous, cream to ferruginous, apex acute. Spathes appressed to scapes, membranaceous, ca. 0.6−1.0 cm long, lamina glabrescent, oblique opening, margins lacerate, ciliate. Scapes free, ca. 4−150 per plant, ca. 6.0−10.0 cm long, filiform, pilose as the leaves. Capitula 3.0−7.0 mm diam. × 3.0−4.0 mm high. Involucral bracts in 4−6 series, ovate-triangular, ca. 2.0−3.0 mm long × 1.0−2.0 mm wide, straw-coloured, darker on the margins, pilose on abaxial surface, mainly in the upper back, ciliate, tufted at apex, trichomes cream, occasionally yellowish at the apex, glabrous on adaxial surface, apex acute to obtuse. Floral bracts lanceolate, membranaceous, ca. 2 mm long, cream at the base, light-brown at the apex, pilose on abaxial surface, ciliate on the margins, cilia shortening toward the obtuse and tufted apex, filamentous trichomes ca. 7−9 cells long, cream, occasionally the distal trichomes yellowish at the apex, glabrous on adaxial surface, apex obtuse. Flowers 3-merous, ca. 60 per capitulum. Staminate flowers ca. 2.0−2.5 mm long; pedicel ca. 0.3 mm long, densely pilose, with filamentous trichomes ca. 1.5 mm long; sepals free, oblanceolate, membranaceous, ca. 1.5−2.0 mm long, cream to straw-coloured, darker at the obtuse apex, pilose as the floral bracts; corolla tubular, apex with tree acute lobes, soon involute, membranaceous, ca. 1.5−2.0 mm long, hyaline, glabrous; stamens ca. 2 mm long, filaments adnate to corolla on its lower third, anthers cream; pistillodes 3, ca. 0.8 mm long, fimbriate at the apex. Pistillate flowers ca. 2.0−3.0 mm long, pedicel ca. 0.3 mm long, densely pilose, with filamentous trichomes ca. 1.5 mm long; sepals free, oblanceolate, membranaceous, thickening during fruit maturation, hygroscopic, ca. 1.5−2.0 mm long, cream to light-brown, darker at the upper part, pilose as the floral bracts, apex cuspidate; petals free, oblanceolate, membranaceous, ca. 1.5−2.5 mm long, cream to light-brown at the apex, ciliate, tufted at the truncate apex; staminodes 3, scale-like; gynoecium ca. 2.0−3.0 mm long, stigmatic branches ca. 1.5 mm long, bifid, twice longer than the papillose nectariferous branches. Fruit a loculicidal capsule.

**Figure 1.**
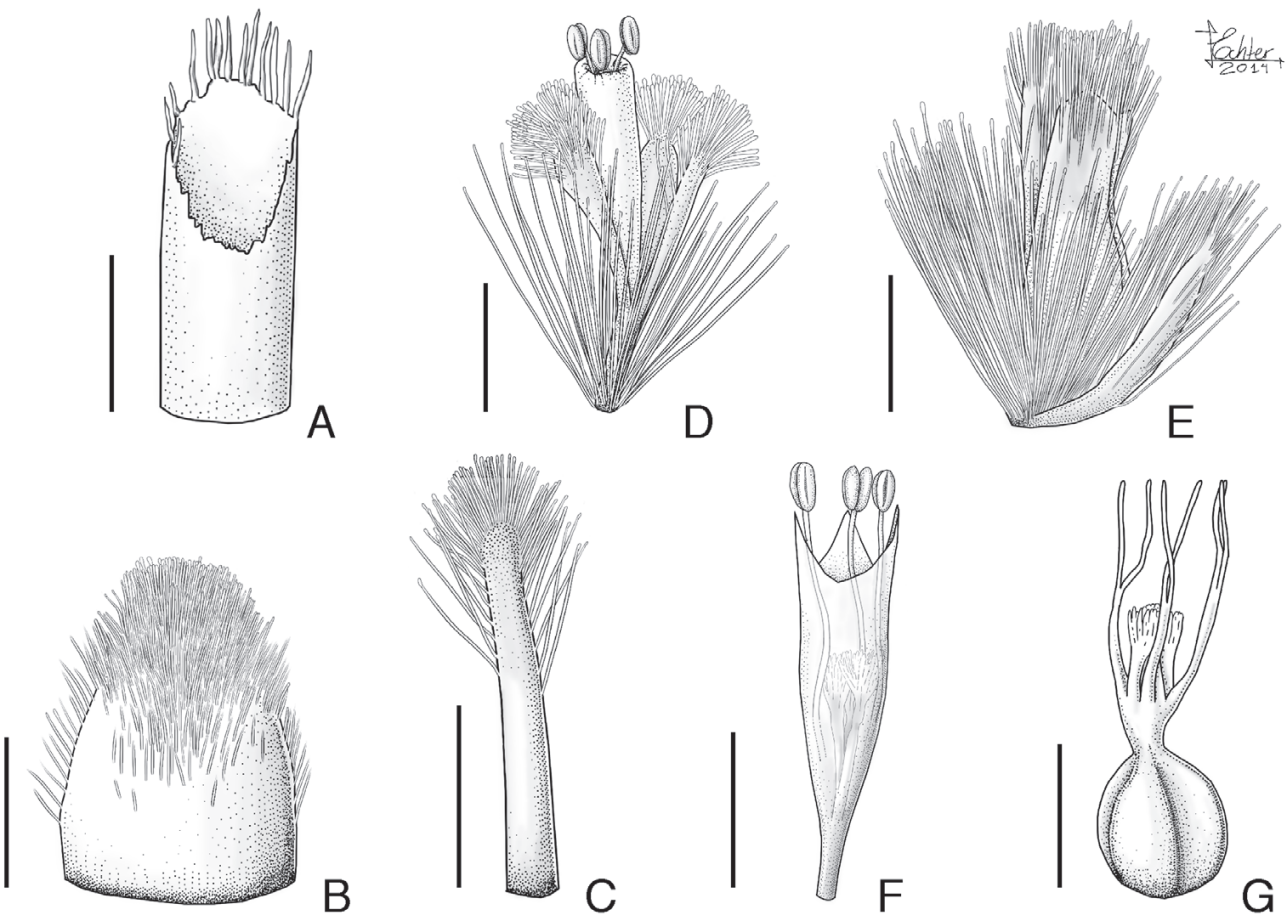
Illustration of *Paepalanthus serpens* Echtern. & Trovó: **A** Spathe apex **B** Involucral bract abaxial surface **C** Floral bract abaxial surface **D** Staminate flower **E** Pistillate flower with floral bract **F** Staminate flower with pedicel and sepals removed, at early anthesis **G** Gynoecium (Drawn from the holotype by L. Echternacht).

**Figure 2.**
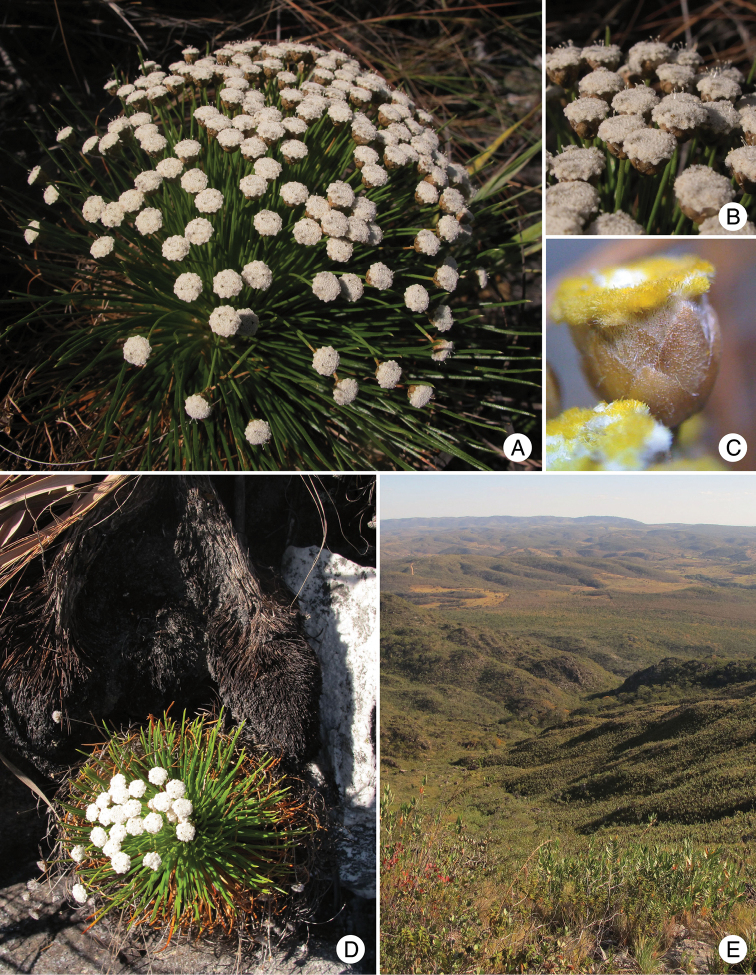
*Paepalanthus serpens* Echtern. & Trovó: **A** Habit **B** Capitula **C** Capitulum with yellowish hairs in a dried individual **D** Habit showing the elongate, thick and creeping stem, with erect apex **E** Habitat (Photos by L. Echternacht).

#### Additional specimens examined.

Brazil. Minas Gerais: Santana do Pirapama, Trilha da Captação, 19°00'30.7"S, 43°45'53.1"W, 840 m, 8 Sep 2011, *C. A. Ferreira Júnior s.n.* (BHZB 8198); same locality, 19°00.52'S, 43°75.85'W DD, 854 m, 19 Mar 2011, *W. Milliken et al. 4296* (SPF, K).

#### Distribution and ecology.

*Paepalanthus serpens* is known from one population at the western slopes of the Serra do Cipó, southern Espinhaço Range, in Minas Gerais state. The habitat consists of a *campo rupestre* within the Cerrado biome. The species grows on gravelly soils of quartzite origin, on open areas, among herbaceous to shrubby vegetation. The encountered population consists of around 300 individuals.

#### Phenology.

Individuals flower during the dry season, starting to develop the inflorescences in March and dispersing seeds until October. The sepals of the pistillate flowers are thickened and hygroscopic when fruits are mature, becoming revolute upon drying, probably favouring seeds dispersal through a catapult mechanism (further description of this dispersal mechanisms is provided by Trovó and Stützel 2012).

#### Conservation status.

The species is considered critically endangered according to criteria B1a and B2a of the IUCN (2011). However, it occurs inside a conservation unit (RPPN Toucan Cipó) and is conserved *ex situ* at the Belo Horizonte Botanical Garden (Fundação Zoo-Botânica de Belo Horizonte, FZB-BH), factors that may attenuate its threatened status.

#### Etymology.

The epithet *serpens* refers to the serpent-like habit of the perennial individuals, which have an unbranched, thick woody stem that slowly elongates and becomes creeping, with an erect apex.

#### Comments.

The extremely reduced population and restricted occurrence range probably contributed to this species remaining undescribed. It occurs on high slopes with difficult access from the closest trails and roads.

The species architecture and trimerous flowers place *Paelanthus serpens* within Paelanthus ser. Paepalanthus (Ruhland 1903), which is the largest of the genus, encompassing most of its morphological, taxonomic and phylogenetic complexity (Andrade et al. 2010, Giulietti et al. 2012, Trovó et al. 2013). Usually species within this series are hard to identify (Andrino 2013), making the confident recognition of new species challenging. *Paepalanthus serpens*, however, is very different from the other species of the series in several aspects. It can be easily distinguished by its robust habit, with an elongate, lignescent stem, thickened by marcescent leaf sheaths, bearing at the apex a rosette of linear and erect leaves, numerous scapes equalling the leaf height and capitula with straw-coloured involucre. This character set is unique within the genus. Other peculiar features are the scape spathes, which are quite short (up to 1 cm) and membranaceous, with lacerate margins, and short ferrugineous trichomes on the leaves and scapes. In addition, in some individuals, the trichomes on the bracts and flowers become yellowish after drying, which is an unusual feature within the genus.

The species of *Paepalanthus* with similar overall morphology to *Paepalanthus serpens* are *Paepalanthus caespititius* Mart. ex Koern. and *Paepalanthus brunnescens* Ruhland. Both have a similar size and linear leaves, but more delicate habit and short, non-lignescent stem. These species are also not sympatric to *Paepalanthus serpens*, as none of them are reported to the Serra do Cipó. *Paepalanthus caespititius* is morphologically the most similar species, with similar leaf width and short scapes, surpassing the leaves by no more than 5 cm. However, it shows dark involucral bracts and lanose stem with long ferrugineous hairs. *Paepalanthus brunnescens* resembles *Paepalanthus serpens* by its straw-coloured capitulum involucre, but can be easily differentiated by its short, branched stem, scapes greatly surpassing the leaf height, larger leaves, and elliptic to lanceolate involucral bracts.

## Supplementary Material

XML Treatment for Paepalanthus serpens
